# Epidemiology of outpatient and inpatient eye injury in Taiwan: 2000, 2005, 2010, and 2015

**DOI:** 10.1371/journal.pone.0235208

**Published:** 2020-07-01

**Authors:** Jiahn-Shing Lee, Wei-Min Chen, Lu-Hsiang Huang, Chia-Chi Chung, Kuang-Hui Yu, Chang-Fu Kuo, Lai-Chu See

**Affiliations:** 1 Department of Ophthalmology, Chang Gung Memorial Hospital & College of Medicine, Chang Gung University, Taoyuan, Taiwan; 2 Department of Public Health, College of Medicine, Chang Gung University, Taoyuan, Taiwan; 3 Division of Rheumatology, Allergy and Immunology, Chang Gung Memorial Hospital at Linkou, Taoyuan, Taiwan; 4 Biostatistics Core Laboratory, Molecular Medicine Research Center, Chang Gung University, Taoyuan, Taiwan; 5 Center for Big Data Analytics and Statistics, Chang Gung Memorial Hospital at Linkou, Taoyuan, Taiwan; Universita degli Studi dell'Insubria, ITALY

## Abstract

**Purpose:**

To estimate the incidence rate of eye injuries (EI) requiring inpatient and outpatient treatment in Taiwan and compare the epidemiologic characteristics of EI (age, sex, treatment setting, seasonality, occupation, external cause, diagnosis, and surgery) in the years 2000, 2005, 2010, and 2015.

**Methods:**

We analyzed four random samples of 1,000,000 beneficiaries each from 2000, 2005, 2010, and 2015 of the Taiwan National Health Insurance Program. The direct age-standardized rate, with 95% confidence interval (CI), was used to compare EI rates for the four calendar years. The chi-square test and chi-square test for trend were used to compare data for the four calendar years.

**Results:**

Annual EI incidence rates were between 2.57% in 2000 and 3.28% in 2015. The age-standardized rates were 2.73% (95% CI, 2.70%–2.76%) in 2000, 3.37% (95% CI, 3.33%–3.41%) in 2005, 3.31% (95% CI, 3.28%–3.35%) in 2010, and 3.02% (95% CI, 2.99%–3.06%) in 2015. Manual workers had the highest EI incidence rate, followed by non-manual workers and civil servants. The proportion of EI requiring inpatient treatment declined from 1.34% in 2000 to 0.63% in 2015 (*P* <0.0001). Analysis of seasonality showed a consistent decrease in February in the four sampling years; however, this decrease in EI was only seen in outpatients, not in EI requiring hospitalization. The proportion of outpatients requiring surgery significantly decreased, from 2.53% in 2000 to 1.2% in 2015 (P<0.0001). However, the proportion of inpatients requiring surgery for EI as the principal diagnosis increased from 69.32% in 2000 to 83.02% in 2015 (P = 0.29), and the proportion of inpatients requiring surgery for EI as a secondary diagnosis increased from 54.86% in 2000 to 71.6% in 2015 (P = 0.0019). Among inpatients with EI, the most common cause of EI was a traffic accident (44.79%, especially motorcycles), followed by falls (9.75%) and homicide (6.05%).

**Conclusion:**

In Taiwan, the annual EI incidence rate slightly increased from 2000 to 2005 and then decreased through 2015. The proportion of EI patients requiring hospitalization decreased from 1.34% in 2000 to 0.63% in 2015, but the percentage of inpatients requiring surgery increased. Traffic accidents (especially those involving motorcyclists) remained the predominant external cause of EI requiring hospitalization during the study period.

## Introduction

In 1998, the World Health Organization (WHO) published a review of the global impact of eye injuries (EI). The report compiled data from the WHO Blindness Data Bank and 54 published or unpublished reviews/reports from 1971 to 1995, most of which were from higher-income countries [1]. The report noted that (1) 55 million EI that restricted activity for longer than one day occurred each year, (2) 750,000 persons required hospitalization each year for EI, including some 200,000 with open-globe injuries, and (3) about 1.6 million were blinded by injuries, 2.3 million had poor bilateral vision after EI, and almost 19 million had unilateral blindness or low vision after EI. The WHO requested that future epidemiologic studies use a standardized international template for reporting EI, to ensure accurate estimates of EI incidence, prevalence, and impact. However, almost two decades after that review was published, many countries have not yet provided detailed epidemiologic data on EI. A recent systematic review of ocular trauma registries proposed the development of consensus guidelines for an international ocular trauma registry that includes the mechanism and context of injury and visual outcomes [2].

EI severity can be graded based on a treatment level, as follows: (1) self/home management, (2) treatment in an outpatient or emergency department, followed by release, (3) hospitalization, and (4) use of rehabilitation services [3]. In population-based and nationwide studies of patients with EI as the principal discharge diagnosis, the incidence rate ranged from 9.8 [4] to 13.2 [5–7] per 100,000 person-years. When EI was defined as any discharge diagnosis of EI, the incidence rate ranged from 27.3 [5, 6] to 35.3 [4] per 100,000 person-years.

Although less-severe EI do not typically require hospitalization and are less a threat to sight, they are much more frequent and therefore warrant comprehensive epidemiologic investigation. In a study using telephone interviews, the incidence rate of EI among US adults in New England was 975 per 100,000 persons, much higher than the rate of EI requiring hospitalization [8]. A similar study of data collected from 2009 through 2011 in Singapore found that ocular trauma affected 1 in 25 adults older than 40 years, 20% of whom required hospitalization [9]. From 2006 through 2011, there were 5,541,434 visits to emergency departments in the United States for a principal or other diagnosis of ocular trauma, a substantial number of which were emergency department cases [10].

In this study, we used nationwide samples comprising 4 million persons (1 million each year) in calendar years 2000, 2005, 2010, and 2015 to estimate the rate of EI requiring inpatient and outpatient treatment in Taiwan and compare the epidemiologic characteristics of EI (age, sex, treatment setting, seasonality, occupation, external cause, diagnosis, and surgery) in those years.

## Methods

### Data source

In 1995, Taiwan launched a compulsory single-payer National Health Insurance (NHI) system, which covered 99.9% of the population by 2018 [11]. The National Health Insurance Research Database (NHIRD) contains registration files and original claims data and is released for research purposes. Four Longitudinal Health Insurance Databases (LHIDs) were constructed: the LHID2000, LHID2005, LHID2010 and LHID2015. For LHID2000, the National Health Research Institute (NHRI) randomly sampled 1,000,000 persons from all beneficiaries of the NHI program during the period 1996–2000. For LHID2005 and LHID2010, the NHRI drew random samples of 1,000,000 persons from all beneficiaries of the NHI program in 2005 and 2010, respectively. For LHID2015, we drew a random sample of 1,000,000 persons from all beneficiaries of the NHI program in 2015 by using the same algorithm (SAS statements) as the NHRI. There were 21.4 million (M), 22.31 M, 23.07 M, and 23.74 M beneficiaries in 2000, 2005, 2010, and 2015, respectively [12]. Hence, the sampling rate was between 4.21% and 4.67% for the four sampling years. All data on registration, outpatient claims, and inpatient claims from the quarter before the sampling year until the quarter after the sampling year for these four samples of 1,000,000 persons were included in LHID2000, LHID2005, LHID2010, and LHID2015, respectively. The section "Ascertainment of eye injury", below, explains why each LHID covered the period starting one quarter before the sampling year and ending one quarter after the sampling year. There was no significant difference in sex distribution between patients in the LHID and those in the original NHIRD (χ^2^  = 1.74, df  = 1, *P*  = 0.19 for LHID2000; χ^2^  = 0.008, df  = 1, *P*  = 0.93 for LHID2005; χ^2^  = 0.067, df  = 1, *P*  = 0.80 for LHID2010; χ^2^  = 0.956, df  = 1, *P* = 0.33 for LHID2015) [13].

The primary data source for this study were the LHID2000, LHID2005, LHID2010, and LHID2015 databases. This study obtained an exemption from the Institutional Review Board of Chang Gung Memorial Hospital, Taiwan (101-4675B), because all data were completely anonymous. The study adhered to the principles of the Declaration of Helsinki.

### Study design

This cross-sectional study analyzed data from years 2000, 2005, 2010, and 2015. Although the data resemble a longitudinal cohort, we only used data for the year 2000 from LHID2000, data for the year 2005 from LHID2005, data for the year 2010 from LHID2010, and data for the year 2015 from LHID2015, to avoid the aging problem over time for a fixed cohort [14] and immortal bias of the data before the years of random sampling for the four LHIDs [15].

### Ascertainment of eye injury

**Fig 1:**
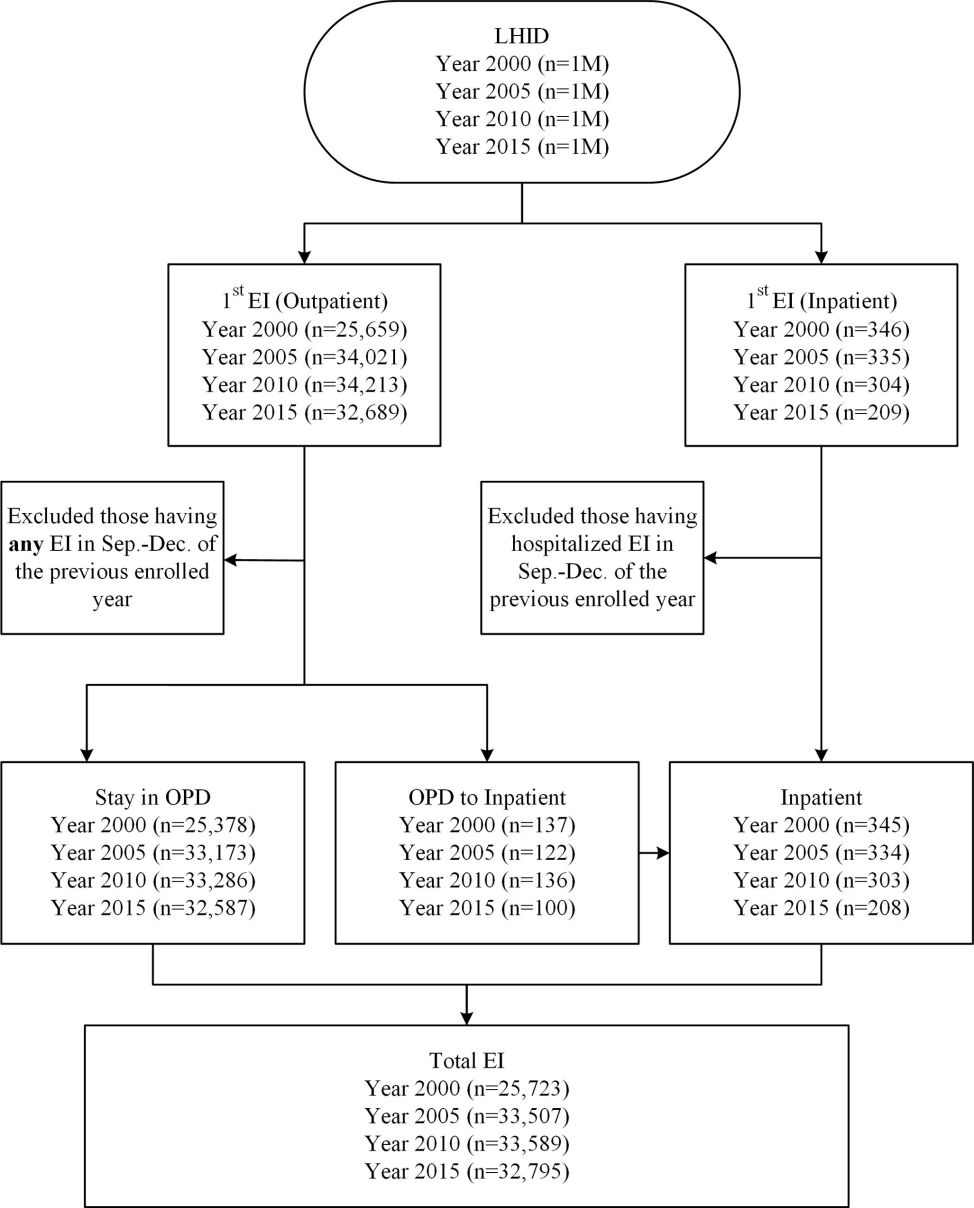
Flowchart for obtaining the study sample. Abbreviations: 1M, 1 million; EI, eye injury; LHID, longitudinal health insurance database; OPD, outpatient department.

We identified records with a diagnosis of EI (International Classification of Diseases, Ninth Revision, Clinical Modification [ICD-9-CM] codes 802.6–802.7, 870.0–870.9, 871.0–871.9, 918.0–918.9, 921.0–921.9, 930.0–930.9, 940.0–940.9, 950.0–950.9, 951.0–951.1, and 951.3) for inpatients and outpatients treated in an outpatient department or emergency department during the years 2000 (from LHID2000), 2005 (from LHID2005), 2010 (from LHID2010), and 2015 (from LHID2015). For EI requiring hospitalization, a principal EI diagnosis was defined as any record with a first diagnosis corresponding to one of the above ICD-9-CM codes. A secondary EI diagnosis was defined as any record in which any of the four secondary diagnoses included one of the above ICD-9-CM codes. Among the very small number of patients who had multiple EI episodes, only the first EI in each four calendar years was included in this study. In other words, the patient, not the episode, with EI was the unit counted. To ensure ascertainment of inpatients with EI, persons with two EI diagnoses (one of which required hospitalization) within three months—the first in an outpatient department and the second as an inpatient, for example—were defined as inpatients with EI, because most inpatients with EI were admitted through an outpatient department or emergency department. Among those with EI not requiring hospitalization, those with an EI during the last three months of the previous calendar year who were treated again in the study calendar year were excluded (Fig 1).

### Covariates

We examined if EI incidence rate was associated with age, sex, seasonality, treatment setting, and occupation. Occupation was divided into four categories: civil servants (full-time or regularly paid personnel with a government or public affiliation), non-manual workers (employees of privately owned institutions), manual workers (self-employed individuals, other employees, and members of farmers' or fishermen's associations), other (veterans, members of low-income families, and substitute service draftees).

### Statistical analysis

Annual incidence rate was calculated as the number of EI in each calendar year divided by the sampled NHI beneficiaries in years 2000, 2005, 2010, and 2015. The 95% confidence interval (CI) of the incidence rates was calculated, after assuming a Poisson distribution. Direct age-standardization with the WHO standard population [16] was used to compare the four incidence rates. The sex rate ratio was defined as the incidence rate for males divided by the incidence rate for females. The chi-square test and chi-square test for trend were used to compare data over the four calendar years.

## Results

### EI rate

Among the four samples of 1 million, EI was diagnosed in 25,723 patients in 2000, in 33,507 in 2005, in 33,589 in 2010, and in 32,795 in 2015 (Fig 1). The annual rate of EI was 2.57% (95% CI, 2.54%–2.60%) in 2000, 3.35% (95% CI, 3.32%–3.39%) in 2005, 3.36% (95% CI, 3.32%–3.39%) in 2010, and 3.28% (95% CI, 3.24%–3.31%) in 2015. Direct age-standardization with the WHO standard population was used to address the difference in age composition of the populations in the four calendar years. The age-standardized rates were 2.73% (95% CI, 2.70%–2.76%) in 2000, 3.37% (95% CI, 3.33%–3.41%) in 2005, 3.31% (95% CI, 3.28%–3.35%) in 2010, and 3.02% (95% CI, 2.99%–3.06%) in 2015.

**Fig 2:**
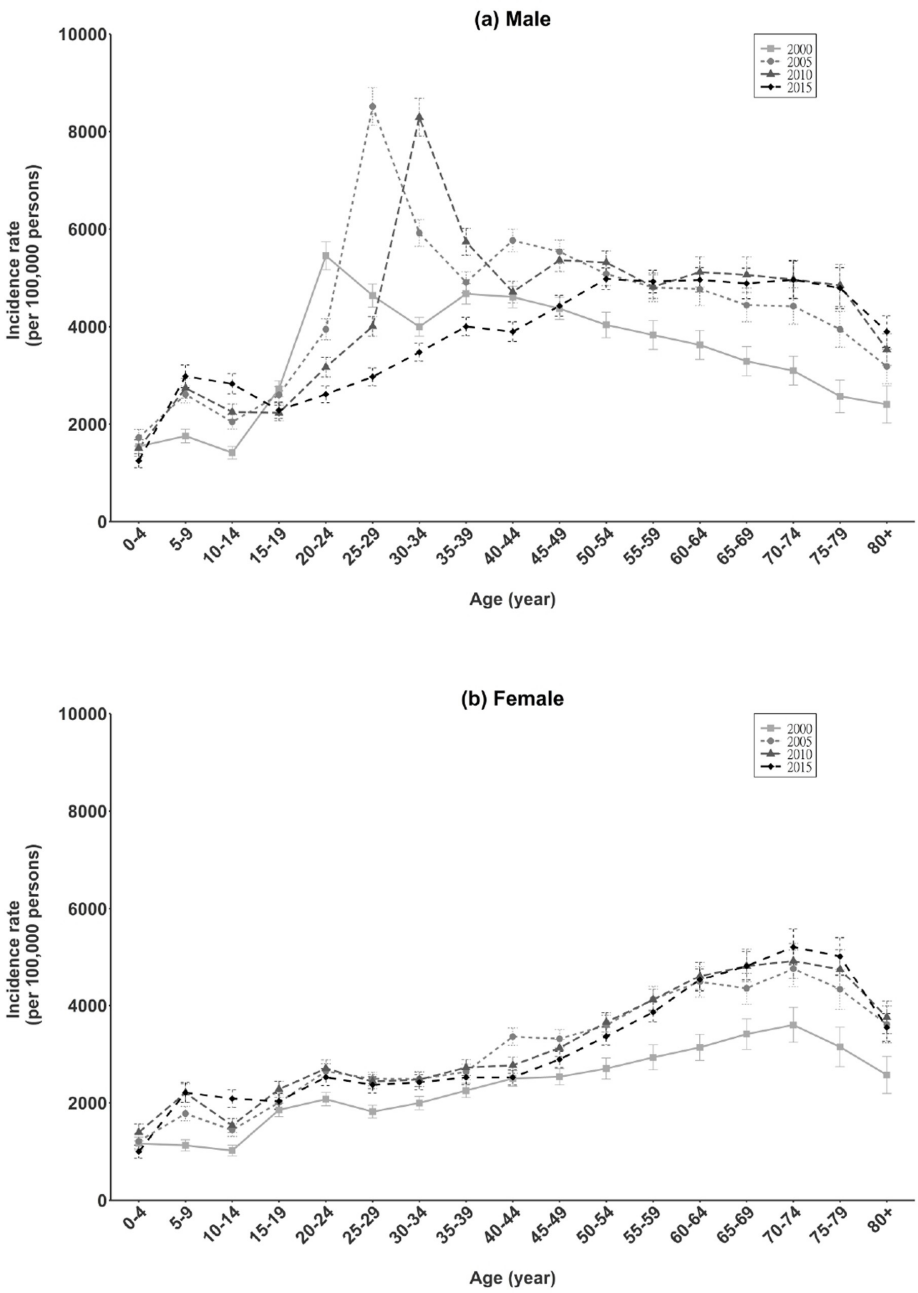
Age-specific incidence rate of total eye injury in Taiwan (2000, 2005, 2010, 2015).

The age-specific rates of EI for males and females are shown in Fig 2A and 2B, respectively. The male/female rate ratio for EI was 1.63 (95% CI, 1.60–1.66) in 2000, 1.41 (95% CI, 1.38–1.44) in 2005, 1.29 (95% CI, 1.27–1.32) in 2010, and 1.24 (95% CI, 1.22–1.26) in 2015. The EI rate was very low for children and young adolescents but increased sharply after age 15 years. During adulthood, the trends for males and females differed. For males, the EI rate was highest at age 20–34 years and decreased thereafter. For females, the EI rate increased continuously until age 70–74 years and declined thereafter.

### Outpatients and inpatients

Most EI patients were treated at outpatient clinics, and the proportion of such patients increased from 98.66% in 2000 to 99.37% in 2015. Hence, the proportion of inpatients with EI decreased from 1.34% in 2000 to 0.63% in 2015 (*P* <0.0001) (Table 1).

**Table 1:** Treatment place and number of eye injury–related diagnoses for patients with eye injury in Taiwan (2000, 2005, 2010, 2015).

	Total (n = 125,614)		2000 (n = 25,723)		2005 (n = 33,507)		2010 (n = 33,589)		2015 (n = 32,795)		p
**Outpatient**	**124,424**	**99.05%**	**25,378**	**98.66%**	**33,173**	**99.00%**	**33,286**	**99.10%**	**32,587**	**99.37%**	<0.0001^1^
Ophthalmic department	115,286	92.66%	22,556	88.88%	30,586	92.20%	31,319	94.09%	30,825	94.59%	<0.0001^2^
Other department	6,302	5.06%	2,669	10.52%	1,910	5.76%	973	2.92%	750	2.30%	
ED department	2,836	2.28%	153	0.60%	677	2.04%	994	2.99%	1012	3.11%	
**Inpatient**	**1,190**	**0.95%**	**345**	**1.34%**	**334**	**1.00%**	**303**	**0.90%**	**208**	**0.63%**	
Principal diagnosis	330	27.73%	88	25.51%	98	29.34%	91	30.03%	53	25.48%	0.46^3^
Secondary diagnosis	860	72.27%	257	74.49%	236	70.66%	212	69.97%	155	74.52%	
**Number of eye injury-related diagnosis**											
**Outpatient**											<0.0001^4^
1	117,522	94.45%	24,485	96.48%	31,595	95.24%	31,143	93.56%	30,299	92.98%	
2	6,741	5.42%	883	3.48%	1,551	4.68%	2,074	6.23%	2,233	6.85%	
3	161	0.13%	10	0.04%	27	0.08%	69	0.21%	55	0.17%	
**Inpatient with principal diagnosis of EI**											0.99^4^
1	263	79.70%	70	79.55%	79	80.61%	71	78.02%	43	81.13%	
2	53	16.06%	14	15.91%	14	14.29%	17	18.68%	8	15.09%	
3+	14	4.24%	4	4.55%	5	5.10%	3	3.30%	2	3.78%	
**Inpatient with secondary diagnosis of EI**											0.62^4^
1	760	89.94%	220	90.91%	214	90.68%	188	88.68%	138	89.03%	
2	82	9.70%	20	8.26%	22	9.32%	24	11.32%	16	10.32%	
3+	3	0.36%	2	0.83%	0	0.00%	0	0.00%	1	0.65%	

^1^Chi-square test: outpatient vs. inpatient over 4 calendar years; ^2^Chi-square test: ophthalmic department, other department, and ED department over 4 calendar years; ^3^Chi-square test: principal diagnosis vs. secondary diagnosis over 4 calendar years; ^4^Chi-square test for trend.

Most outpatients with EI were treated at ophthalmic outpatient clinics, and the proportion increased from 88.88% in 2000 to 94.59% in 2015. The proportion of EI patients treated at emergency departments rose from 0.60% in 2000 to 3.11% in 2015. The proportion of EI patients treated at clinics other than ophthalmic departments decreased from 10.52% in 2000 to 2.30% in 2015. The proportions of EI patients treated in ophthalmic departments, non-ophthalmic departments, and emergency departments significantly differed in 2000, 2005, 2010, and 2015 (*P* <0.0001). For inpatients with EI, the proportion of those with a principal diagnosis of EI increased from 25.51% in 2000 to 30.03% in 2010 and then decreased to 25.48% in 2015, but there was no significant difference over the four calendar years (*P* = 0.46) (Table 1).

### Seasonality

**Fig 3:**
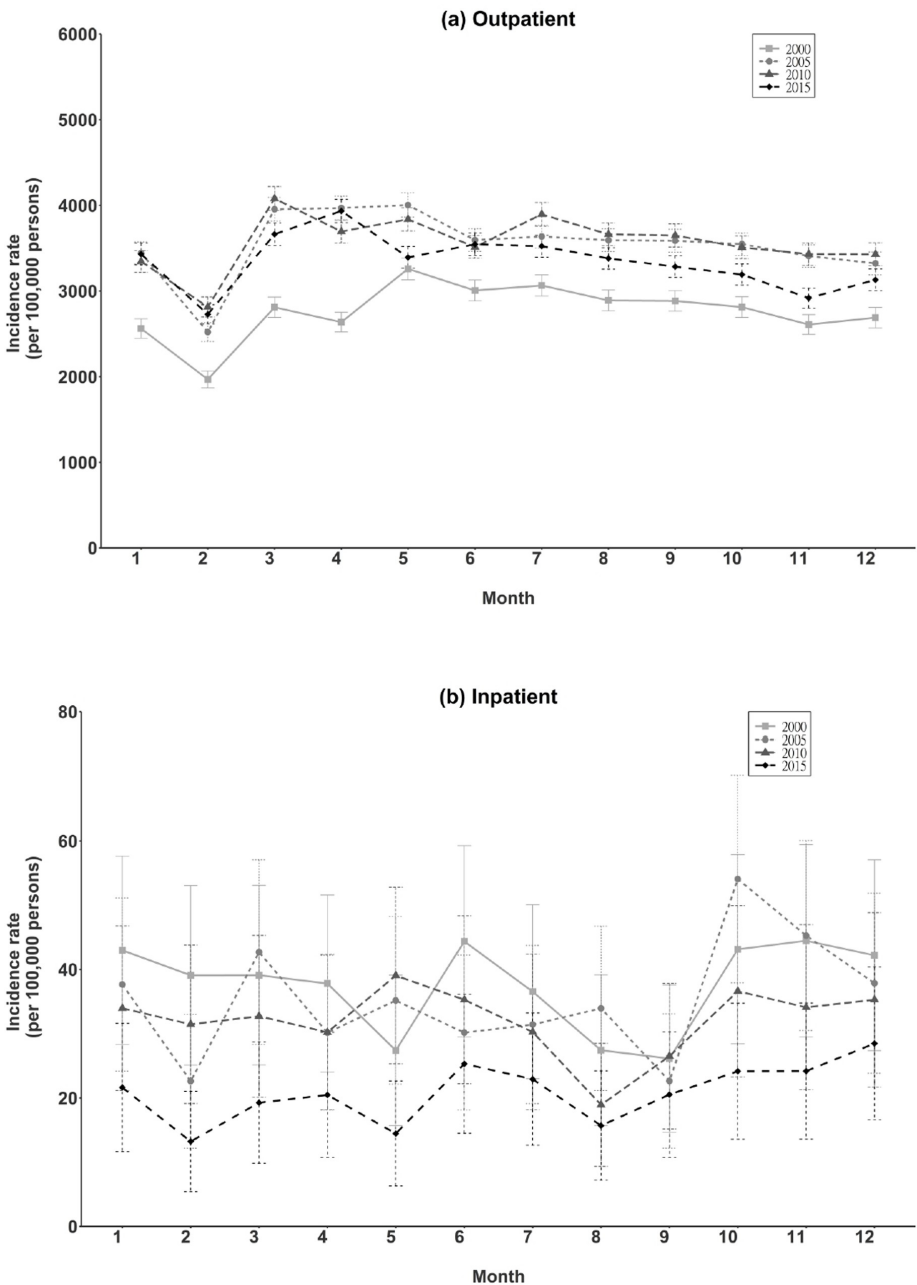
Month-specific incidence rate of total eye injury in Taiwan (2000, 2005, 2010, 2015).

When EI incidence rate was analyzed by month, it was consistently lowest in February, among those treated at outpatient clinics, in all four calendar years (Fig 3A). This trend was not observed among persons treated as inpatients (Fig 3B).

### Occupation

**Fig 4:**
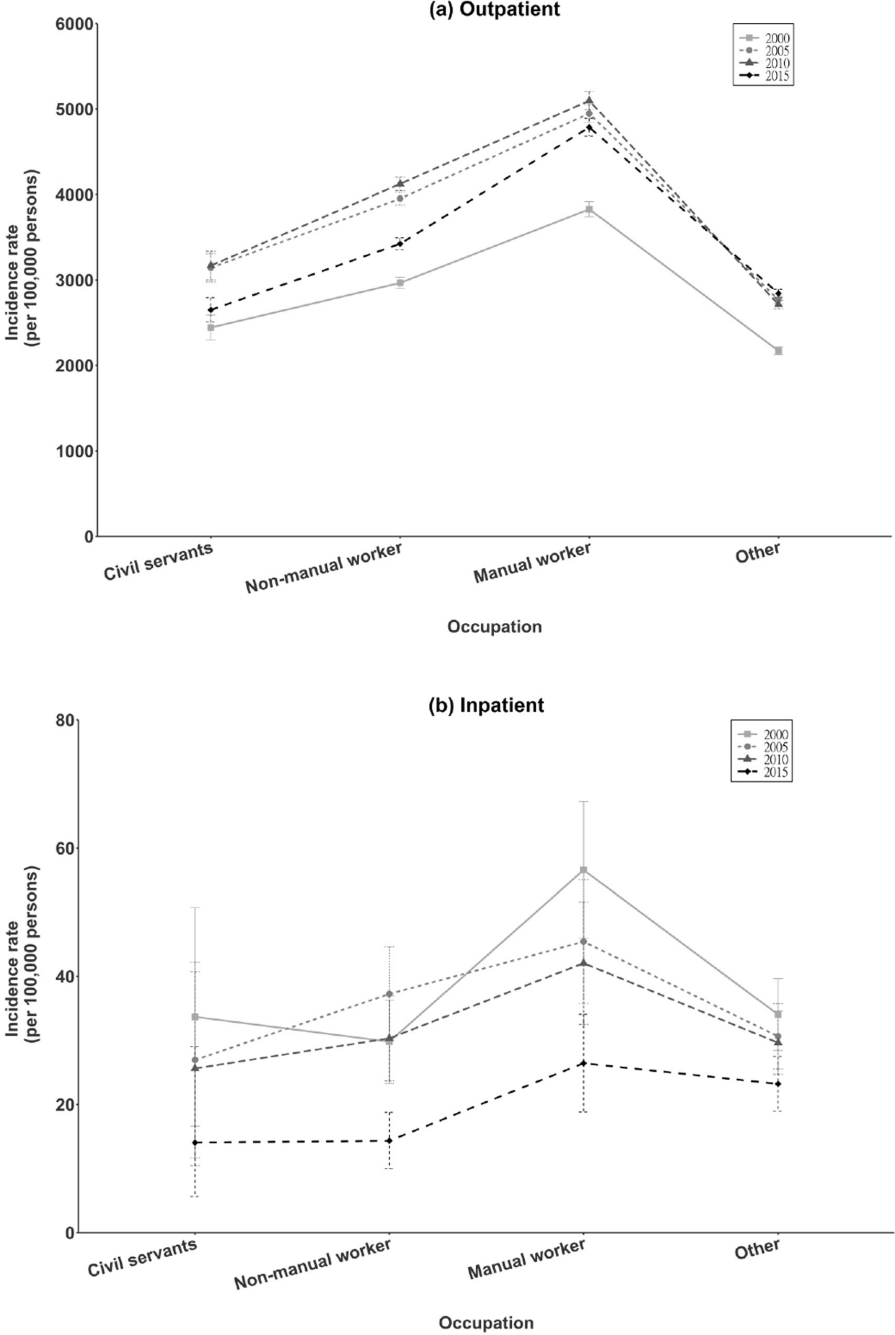
Occupation-specific incidence rate of total eye injury in Taiwan (2000, 2005, 2010, 2015).

Analysis of EI incidence rate in relation to occupation showed that, among outpatients (Fig 4A) and inpatients (Fig 4B), manual workers had the highest rate, followed by non-manual workers and civil servants.

### Diagnosis

**Fig 5:**
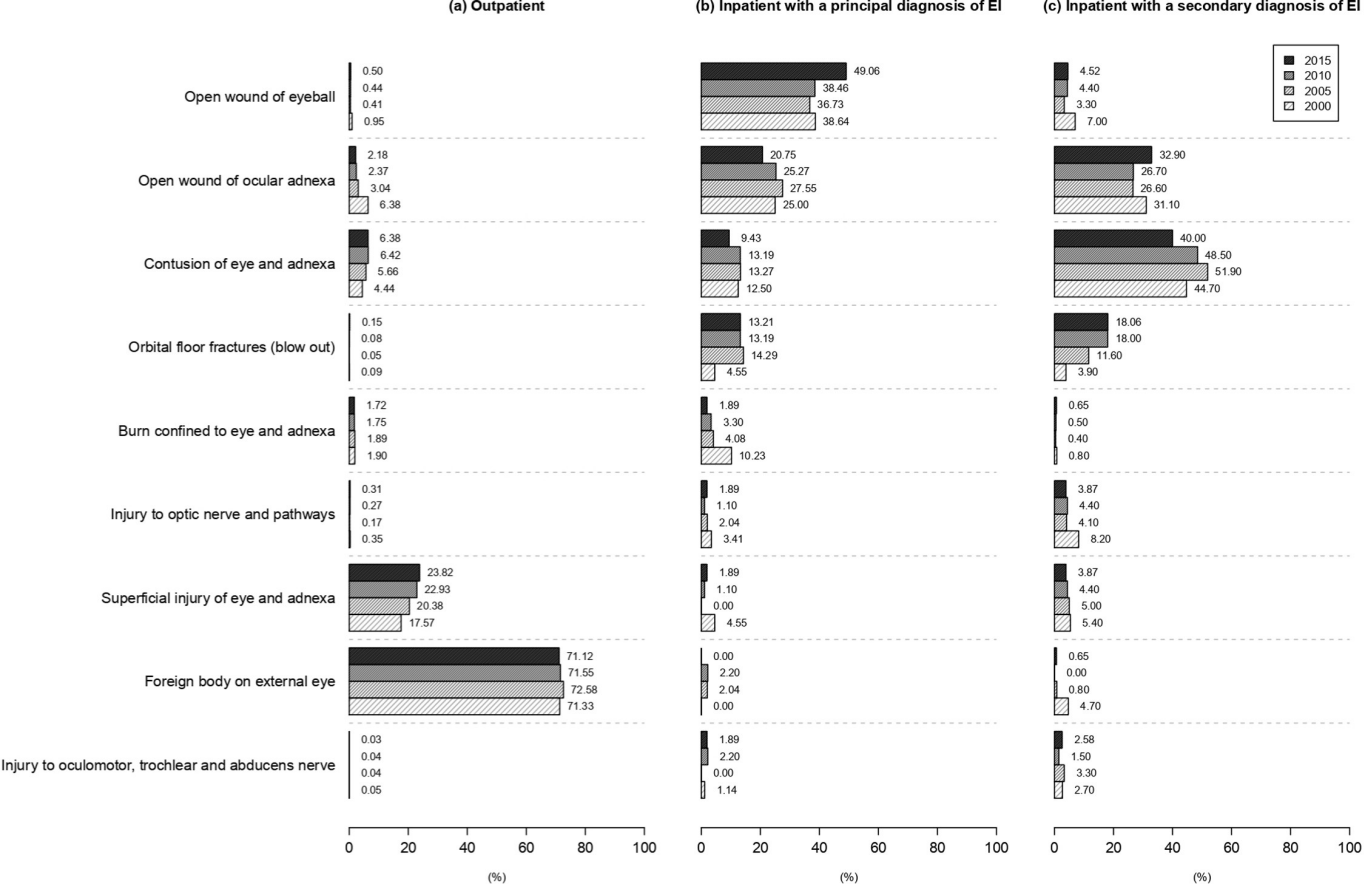
Eye injury–related diagnosis, by treatment place and diagnosis, for total eye injuries in Taiwan (2000, 2005, 2010, 2015).

Among outpatients, the percentage of patients with only one EI diagnosis was 96.48% in 2000 and gradually decreased to 92.98% in 2015. The percentage of patients with 2 or more EI diagnoses gradually and significantly increased from 2000 (3.52%) to 2015 (7.02%) (Table 1). The most common EI-related diagnoses for outpatients with EI were “foreign body on external eye" (71.33% in 2000, 72.58% in 2005, 71.55% in 2010, and 71.12% in 2015)”, “superficial injury of eye and adnexa” (17.57%, 20.38%, 22.93%, and 23.82%, respectively), “open wound of ocular adnexa" (6.38%, 3.04%, 2.37%, and 2.18%, respectively), and “contusion of eye and adnexa" (4.44%, 5.66%, 6.42%, and 6.38%, respectively) (Fig 5).

Among inpatients with a principal diagnosis of EI, the most common EI-related diagnosis was “open wound of eyeball" (38.64% in 2000, 36.73% in 2005, 38.46% in 2010, and 49.06% in 2015), followed by “open wound of ocular adnexa" (25.00%, 27.55%, 25.27%, and 20.75%, respectively), and “contusion of eye and adnexa" (12.50%, 13.27%, 13.19%, and 9.43%, respectively). This order of incidence was identical in 2000, 2005, 2010, and 2015. The percentage of patients with a diagnosis of “orbital floor fractures” increased from 4.55% in 2000 to 14.29% in 2005 to 13.19% in 2010 to 13.21% in 2015. The percentage of patients with a diagnosis of “burn confined to eye and adnexa” decreased from 10.23% in 2000 to 1.89% in 2015 (Fig 5). Most patients had only 1 EI-related diagnosis (between 79.55% and 81.13%), followed by those with 2 EI-related diagnoses (between 14.29% and 18.68%) and 3+ EI-related diagnoses (between 3.30% and 5.10%). The distributions of patients with 1, 2, or 3+ EI-related diagnoses were similar in the 4 calendar years (*P* = 0.99) (Table 1).

Among inpatients with a secondary diagnosis of EI, the most common EI-related diagnosis was “contusion of eye and adnexa" (44.70% in 2000, 51.90% in 2005, 48.50% in 2010, and 40.00% in 2015), followed by “open wound of ocular adnexa" (31.10%, 26.60%, 26.70%, and 32.90%, respectively), “orbital floor fractures" (3.90%, 11.60%, 18.00%, and 18.06%, respectively), and “injury to optic nerve and pathways" (8.20%, 4.10%, 4.40%, and 3.87%, respectively) (Fig 5). Most patients had only 1 EI-related diagnosis (between 88.68% and 90.91%), followed by those with 2 EI-related diagnoses (between 8.26% and 11.32%). The distributions of patients with 1, 2 or 3+ EI-related diagnoses were similar in the four calendar years (P = 0.62)(Table 1).

### Surgery

In total, 2.07% EI required surgery. However, the proportion of surgical cases was disproportionately distributed among three groups (1.43% for outpatients, 78.49% for inpatients with a principal diagnosis of EI, and 65.46% for inpatients with a secondary diagnosis of EI; *P* <0.0001). Among surgical cases, 65.3% of operations were eye-related. The percentage of outpatients with EI who required surgery declined from 2.53% in 2000 to 1.20% in 2015 (*P* <0.0001) (Table 2).

**Table 2:** Percentage of eye injury patients in Taiwan who required surgery, by treatment place (2000, 2005, 2010, 2015).

	Total (n = 125,614)		2000 (n = 25,723)		2005 (n = 33,507)		2010 (n = 33,589)		2015 (n = 32,795)		P^1^
**Surgery needed**											
**Outpatient**											<0.0001
**None**	1,22,650	98.57%	24,735	97.47%	32,758	98.75%	32,959	99.02%	32,198	98.81%	
**Eye-related**	1,363	1.10%	496	1.95%	374	1.13%	236	0.71%	257	0.79%	
**Non-eye-related**	411	0.33%	147	0.58%	41	0.12%	91	0.27%	132	0.41%	
**Inpatient with principal diagnosis of EI**											0.29
**None**	71	21.52%	27	30.68%	18	18.37%	17	18.68%	9	16.98%	
**Eye-related**	211	63.94%	52	59.09%	66	67.35%	59	64.84%	34	64.15%	
**Non-eye-related**	48	14.55%	9	10.23%	14	14.29%	15	16.48%	10	18.87%	
**Inpatient with secondary diagnosis of EI**											0.0019
**None**	297	34.53%	116	45.14%	78	33.05%	59	27.83%	44	28.39%	
**Eye-related**	120	13.95%	33	12.84%	34	14.41%	33	15.57%	20	12.90%	
**Non-eye-related**	443	51.51%	108	42.02%	124	52.54%	120	56.60%	91	58.71%	

^1^Chi-square test

Among inpatients with a principal diagnosis of EI, the percentage requiring surgery increased from 69.32% in 2000 to 83.02% in 2015, but the difference was not significant (*P*  = 0.29). Among inpatients with a secondary diagnosis of EI, the percentage requiring surgery significantly increased from 54.86% in 2000 to 71.61% in 2015 (*P*  = 0.0019); 63.94% of surgeries were eye-related among those with a principal diagnosis of EI, and 13.95% of surgeries were eye-related among those with a secondary diagnosis of EI (Table 2).

### External causes of EI

**Fig 6:**
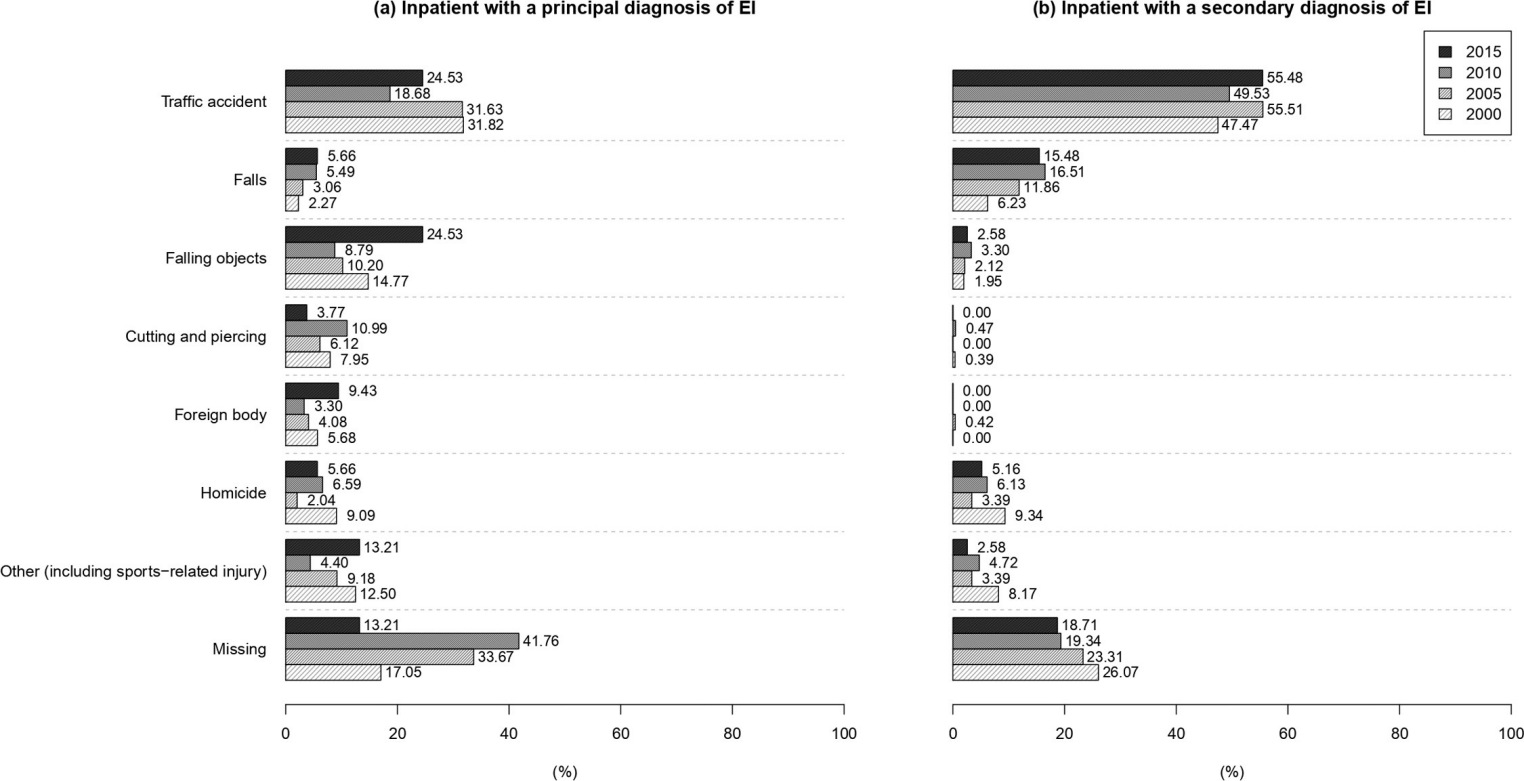
External causes of eye injuries requiring hospitalization in Taiwan (2000, 2005, 2010, 2015).

Among inpatients with EI, E-codes or external causes of injuries were available for 237 (71.82%) with a principal diagnosis of EI and for 668 (77.67%) with a secondary diagnosis of EI. The most common cause among inpatients with EI was “traffic accident" (26.97% of inpatients with a principal diagnosis of EI; 51.63% of those with a secondary diagnosis of EI). The second and third most common causes were “accidentally hit by falling objects" (13.33%) and “accidents caused by cutting or piercing objects" (7.58%) for inpatients with a principal diagnosis of EI, and “accidental fall" (11.98%) and “homicide (including rape, or child battering)" (6.16%) for those with a secondary diagnosis of EI. Among patients with “traffic accidents”, motorcycle accidents were the most common cause of EI for those with a principal (12.12%: 12.50% in 2000, 11.22% in 2005, 13.19% in 2010, 11.32% in 2015) and secondary (30.93%: 21.79% in 2000, 33.47% in 2005, 34.43% in 2010, 37.42% in 2015) diagnosis of EI. The distributions of E-codes or external causes among inpatients with EI as a principal diagnosis were similar for the four calendar years (P = 0.30). However, the distribution of E-codes or external causes among inpatients with EI as a secondary diagnosis significantly differed among the four calendar years (P = 0.0048). The proportion of cases attributable to falls increased from 2000 until 2015 (P = 0.0025) (Fig 6).

## Discussion

The present population-based sample of all outpatients and inpatients in Taiwan yielded a comprehensive profile of EI in these groups for four calendar years (2000, 2005, 2010, and 2015).

### EI incidence rate, by calendar year

The annual rate of EI was 2.57% in 2000, 3.35% in 2005, 3.36% in 2010, and 3.28% in 2015. The age-standardized incidence rate of EI (2.73%–3.02%) was higher than WHO global estimates (incident EI that restricts activity for longer than 1 day, 0.95%; incident EI requiring medical attention in adults, 1.0%) [1]. The present incidence of EI requiring medical treatment was also higher than rates in the US region of New England in 1985 (9.8 per 1000) [8], in the United States from 1992 through 2001 (6.93 per 1000) [17], and in New Zealand adults during 2007–2016 (10.7 per 1000) [18]. Differences in research methods and EI criteria complicate any comparison between studies of prevalence and incidence rates. Glynn et al. used random digit–dialed telephone interviews to calculate the EI rate in the preceding year [8]. However, underestimation is a potential concern in their study because people tend to forget minor eye problems. McGwin et al. used the National Ambulatory Medical Care Survey, National Hospital Ambulatory Medical Care Survey, and National Hospital Discharge Survey to collect data on EI in the United States during 1992–2001 [17]. The exclusion of optometric care explains the difference in rates between the present study and their report. Furthermore, the high coverage rate (99.6%) of the Taiwan national compulsory insurance program, as well as the convenience of medical service and the affordable copayments, may explained the higher EI rate in this study.

The higher annual rates of EI in 2005 and 2010 are likely attributable to a change in the NHI payment system in Taiwan. In addition, the number of ophthalmic clinics increased during the study period. In Taiwan, a fee for service system was used to reimburse health care providers, starting in 1995; however, a global budget was later used to control increasing medical fees in local clinics (starting in 2001) and medical centers (starting in 2002), and this practice continues today. The average number of clinic visits per person-year in Taiwan increased from 14.7 in 2000 to 15.5 in 2005 and plateaued at 15.6 in both 2010 and 2015. The increasing convenience of medical service in Taiwan may partially explain the higher estimates for EI incidence and average number of clinic visits per person-year during this period. The number of available clinics increased from 18,082 in 2000 to 19,433 in 2005, to 20,691 in 2010, and to 22,177 in 2015 (a 22.6% increase). The number of practicing ophthalmologists in Taiwan was 1329 in 2000, 1494 in 2005, 1663 in 2010, and 1740 in 2015 (a 30.9% increase). In contrast, the population in Taiwan increased by only 5.4%: from 22.3 million in 2000 to 23.5 million in 2015 [19].

### EI requiring hospitalization by calendar year

In this study, the proportion of EI patients requiring hospitalization decreased from 1.34% in 2000 to 0.63% in 2015. A decline in the rate of hospitalization for eye trauma was also noted in the US state of Maryland [5] (25% decline in all diagnoses from 1979 to 1986; a 3.1% reduction annually), in the United States nationally (4.2% decline per year from 1992 to 2001, including office-based physician practices, outpatient clinics, emergency departments, and inpatients) [17], and in Singapore [7] (20% decline in principal diagnoses of EI from 1991 to 1996, a 3.3% reduction annually). Changes in the indications and threshold for hospitalization explain the declines in the United States in 1979–1986 [5] and Singapore in 1991–1996 [7]. The rate of eye trauma also decreased in the United States during 1992–2001 [17]. Awareness of health and safety issues, mandatory use of protective devices during athletic activities [20, 21] and at work [22], and seatbelt use [23] partially explain this reduction [24].

In contrast, the incidence of EI requiring hospitalization increased by 18.3% from 2001 through 2014 in the United States [25]. This increase was predominately attributable to secondary diagnoses of EI (82.9%) related to head injuries and to population aging. The risk of vision loss for most of these injuries (superficial injury of eye and adnexa and foreign body on external eye) would have been low [26]. Admissions with primary EI diagnoses were a much smaller proportion in this report, and the size of such a cohort actually declined, by 23%, over the study period.

In Taiwan, decreases in the rate of hospitalization for eye trauma are likely attributable to greater enforcement of laws mandating the use of protective equipment for motor vehicles and the transfer of many manufacturing facilities to lower-income countries [27]. A law requiring motorcycle helmets was enforced beginning in 1997 [28], including later in 2001 a front-seat seatbelt law [29], and a law requiring child car seats in 2004 [30]. To reduce other forms of EI, such as sports-related eye trauma, we suggest that sports-related protective gear be promoted or mandated in Taiwan.

### EI in outpatients by calendar year

The number of ophthalmologists in Taiwan increased from 2000 to 2015, which might explain the increase in the proportion of patients with EI visiting ophthalmic outpatient clinics (88.88% in 2000, 94.59% in 2015) and the decrease in the proportion of those visiting medical facilities other than ophthalmic departments (from 10.52% in 2000 to 2.30% in 2015). Ophthalmologists using professional and magnifying instruments might be able to perform more-detailed assessments of EI, which could have increased the number of EI-related diagnoses. Moreover, to prevent potential malpractice claims, general practitioners might have elected to refer EI patients to emergency departments or ophthalmologists, which could have increased the number of EI-related diagnoses. The Taiwan NHI requires doctors to code accurate and complete diagnoses, to ensure correct payment, and levies substantial fines for errors.

About 97% of EI patients are treated in outpatient clinics, and 2% are treated in emergency departments; only 1% are admitted to hospitals. In previous studies of health care centers, only 10%–27% of EI patients were treated in outpatient clinics, whereas 38%–65% were treated in emergency departments and 5%–16% were admitted to eye hospitals [3]. Although EI treated in outpatient clinics are less severe, the present high incidence in a nationwide survey is a concern. Even less-severe injuries, such as superficial foreign bodies and corneal abrasions, can lead to vision-threatening complications or cause a substantial socioeconomic burden (including time off from work) for otherwise healthy people. A previous study of US adults aged 43–86 years found that 20%–23% of men and 8%–12% of women had at least one EI episode in their lifetimes [3, 31]. The cumulative prevalence of EI requiring medical attention in adults older than 40 years was around 14.4% in Baltimore, United States, and 21.1% in Melbourne, Australia [31–33]. In addition, nearly 2% of adults reported a new EI within 5 years, and a person with a history of EI had 3–5 times the risk of new ocular trauma during the next 5 years [32].

### Seasonality

EI exhibited seasonality. Data from the US emergency departments from 2006–2013 showed a mean annual peak of ocular injuries between May and July [34]. New Zealand national and regional datasets from 2007–2016 revealed that the warmest months of the year, from late Spring (November) until the end of summer (March), had the highest incidence of ocular injuries [18]. The warmer temperatures and longer daylight hours of the summer months are likely to lead to more time outside participating in potentially risky activities [18, 34]. In South-Central China, a high rate of firework-related ocular injuries occurred during the months near the Chinese New Year [35]. In this study, the EI rate decreased in all four Februarys among outpatients but not inpatients, probably because persons with level 1 EI (self/home management) did not seek treatment in outpatient or emergency departments during the long Chinese New Year holiday, when staffing and resources are limited in medical institutions [36].

### Age and sex

Previous studies identified male sex as a strong risk factor for EI: the male/female rate ratio varied from 1.8 to 8.0 [4–7, 37, 38]. This can mainly be attributed to occupational differences and men’s involvement with more risky tasks than women. In this study, the male/female rate ratio for EI was low: 1.24–1.63. Notably, the high male/female ratios in literature [4–7, 37, 38] were derived from severe or hospitalized EI data, and our study included both outpatient EI data (99%) and inpatient EI data (1%). In this study, the male/female rate ratios for EI were 1.23–1.62 for outpatients and 2.30–3.31 for inpatients, respectively (data not shown). The male/female rate ratios for inpatients in this study are similar to previous studies [4, 5]. The low male/female rate ratios of EI (1.23–1.62) for outpatients is uncertain and deserves further study.

EI can occur at any age and are an important cause of ocular morbidity in children [39]. We noted a small peak in the rate of total EI at age 5–9 years in both sexes (750 per 100,000 person-years for children younger than 15 years). The rate was higher than that in a Nepalese survey for 1995–2000 (300 per 100,000 person-years for children younger than 15 years) [40]. Fortunately, most of the present EI in this age group were treated in outpatient departments (99%) and thus were minor.

After childhood, age-specific EI rates differed between sexes. For males, the EI rate was highest at age 20–24 years in 2000, at age 25–29 years in 2005, at age 30–34 years in 2010, at age 50–54 years in 2015. For females, the highest rate was at age 70–74 in all 4 calendar years. The risk of EI was similar for elderly men and women in this and previous studies [4–7]. Elderly adults are at high risk for EI, and the incidence of open wounds of the eyeball was slightly higher in the present adults older than 75 years (as compared with the US state of Maryland [31] and US general population [6]). We believe that the increased risk of ocular trauma in this age group is attributable in part to an increase in the number of ocular surgical procedures performed on elderly adults [6].

### Occupation

In New Zealand, 13.8% of EI among adults occurred at industrial sites [18]. In South-Central China, the most frequent EI requiring hospitalization was the workplace (39.6%); farmer (49.4%) and worker (23.7%) were the most common occupations affected [35]. In this study, manual workers had the highest EI rate for outpatients and inpatients. Previous studies reported 25.4%–73.7% of EI were work-related [41–47]. Most EI in construction, manufacturing, and agricultural settings result from cutting, welding, drilling, and hammering metal [35].

### External cause

The ICD-9-CM provides an optional, supplementary coding system for classification of external causes of injuries and poisoning, known as E-codes. These codes are derived from information abstracted from medical records and can be used to classify the cause of EI. In Taiwan, E-codes were recorded for hospitalized patients only. In this study, the proportion of records without E-codes decreased from 23.8% in 2000 to 17.3% in 2015. The missing proportion in this study, especially for the year 2015, was much smaller than in three US studies (Maryland: 47.7% of principal diagnoses and 60.8% of secondary diagnoses [5]; US: 76% of principal diagnoses and 74% of all diagnoses [6]; California: 69.1% of principal diagnoses and 63.4% of secondary diagnoses [48]) but larger than in studies of the Chaoshan region of China (10.6%) [46] and southeast Ireland (0.4%) [49]. Efforts to update the E-codes were likely due to the requirements of the Taiwan NHI Administration.

The most common cause of EI requiring hospitalization was “traffic accident”, and motorcycles were the most commonly involved vehicle, which is consistent with 1997–2011 data on facial fracture in the NHIRD (traffic accidents, 55.2%; motorcycle accidents in particular, 31.5%) [50]. Other common external causes of EI requiring hospitalization in Taiwan were “falls”, “homicide”, and “other (including "sports-related injuries)”, which might explain the three abovementioned peaks in the age-specific distribution of EI in Taiwan, i.e., for older adults [50], young men, and children (a low peak) (Fig 2). Because of the aging population in Taiwan (proportion of population aged 65 years or older: 8.62% in 2000, 9.74% in 2005, 10.74% in 2010, and 12.61% in 2015) [19], we also observed an increase in the fall proportion for the four calendar years (Fig 6).

### Surgery

Among patients with EI treated at outpatient departments, the percentage needing surgery gradually decreased from 2000 to 2015. The most common EI-related diagnoses in outpatient department cases were “foreign body on external eye”, “superficial injury of eye and adnexa”, “open wound of ocular adnexa”, and “contusion of eye and adnexa”; thus, the decrease in the percentage of outpatients requiring surgery was probably related to the decreasing incidence of “open wound of ocular adnexa” in Taiwan during 2000–2015. The other three common EI-related diagnoses are less likely to require surgery.

The percentage of inpatients with EI who needed surgery slightly increased from 2000 to 2015. The total number (outpatients and inpatients) of surgical cases actually decreased during this period in Taiwan: from 345 in 2000 to 334 in 2005 to 303 in 2010 to 208 in 2015. These findings are not surprising, because more-severe injuries are more likely to be treated on an inpatient basis.

About 76.8% of operations were eye-related for outpatients needing surgery. Among inpatients needing surgery, 81.5% of operations were eye-related when EI was the principal diagnosis, and 21.3% of operations were eye-related when EI was a secondary diagnosis. Patients with severe trauma, especially those needing surgery, frequently receive combined treatment by surgeons in different specialties. Common EI procedures performed by ophthalmologists were cornea repair (37.2%), cataract extraction (13.8%), scleral repair (9.7%), repair of multiple structures of the orbit and globe (11.2%), and vitrectomy (7.4%) for inpatients with work-related EI [48]. A study of hospitalized EI patients in Singapore reported that the most common surgical procedures were complex repair of open-globe injuries (59.0%), simple repair of open-globe injuries (26.8%), and removal of intraocular foreign bodies (14.2%) [7]. Another study, of Italian inpatients, reported that the procedures for EI included medical therapy and simple repair (including conjunctival, corneal, and scleral wounds; 47.0%), complex repair (including large corneo-scleral wounds, excision of prolapsed uveal tissues, lens removal, and anterior vitrectomy; 37.2%), removal of intraocular foreign bodies from the anterior chamber (8.3%), and removal of intraocular foreign bodies from the posterior chamber (9.0%) [47].

### Prevention of EI

Ocular trauma is a leading cause of blindness worldwide, along with cataract, glaucoma, xerophthalmia, trachoma, and onchocerciasis [51]. The latter three conditions being rare in Taiwan, and the former being important health problems in the country. “Accidents” are often regarded as inevitable, although many EI can be prevented by use of protective eye wear and modification of dangerous environments. Identification of individuals at higher risk will help in the development of educational and preventive measures [26, 52–54].

Use of appropriate eye-protection devices at the workplace, in sports, and while motorcycling has not been routine in most Asian countries, including Taiwan [38]. Although the present rate of hospitalization for EI was similar to the world average, the rate of EI not requiring hospitalization only gradually decreased and was still higher than the average. Therefore, EI prevention remains a challenge in Taiwan. An effective EI prevention systems require a multifactorial approach combining legislation, policies, standards, education, and personal use of eye protection, to limit exposure to ocular hazards [54].

### Strengths and limitations

Because of the representative sample, which encompassed EI from outpatients and inpatients for 2000, 2005, 2010, and 2015, we were able to identify temporal trends in EI rate and a decrease in the proportion of admissions. As compared with other hospital-based studies and trauma registries, the Taiwan NHIRD provides a nationwide estimate of EI rate, which is suitable for comparing past, present, and future data. The presence of occupational information in the NHIRD allowed us to identify the elevated risk of EI among manual workers. The comprehensiveness of diagnoses, especially for EI patients requiring hospitalization, revealed different diagnosis profiles for principal and secondary diagnoses of EI.

However, this study has several limitations. First, the external causes of EI in outpatients could not be studied because the E-code for EI was not available in outpatient data. Second, the temporal trend for total injury rate was not examined because only the body site "eye" was included here. Third, the effect of EI on patient vision could not be assessed because the NHIRD lacks detailed information on visual outcomes. Hence, the rate of vision loss in injured eyes is unknown. Fourth, we could not examine the role of injury site (eg, home, work) because this information is not available in the NHIRD.

## Conclusion

In Taiwan, the annual EI incidence rate slightly increased from 2000 to 2005 and then decreased through 2015. The proportion of EI patients requiring hospitalization decreased from 1.34% in 2000 to 0.63% in 2015, but the percentage of inpatients requiring surgery increased. Traffic accidents (especially those involving motorcyclists) remained the predominant external cause of EI requiring hospitalization during the study period.
